# Cranial fasciitis in children: clinicoradiology features and management

**DOI:** 10.1186/s12887-022-03610-w

**Published:** 2022-09-17

**Authors:** Yonghua Xiang, Siping He, Zhengzhen Zhou, Qing Gan, Ke Jin

**Affiliations:** 1 Department of Radiology, Hunan Children’s Hospital, University of South China, No. 86 Ziyuan Road, Changsha, Hunan 410007 China; 2 Department of Pathology, Hunan Children’s Hospital, University of South China, Changsha, China

**Keywords:** Cranial fasciitis, Children, Tomography, X‐ray computed, Management

## Abstract

**Background:**

Cranial fasciitis (CF) is a rare benign fibroproliferative lesion of the skull. To date, the summarized radiologic characteristics and the subtype of the disease have not been reported. our purpose was to summarize the characteristic clinicoradiology features and management of CF and to improve the knowledge of radiologists and clinicians.

**Methods:**

We searched our institution’s database and retrieved the clinical and radiologic data of CF patients confirmed by histopathological examination. The clinicoradiology features and management of CF were analysed retrospectively.

**Results:**

A total of 14 CF patients were included. A total of 85.7% of the patients presented with a painless, firm, nonmobile and single mass. Tenderness and multiple masses were found in 14.3% of the patients. The mass was clearly increased in 2 patients and gradually increased in 5 patients in the short term. We divided these patients into three types based on the CT characteristics. The characteristic features of type I (9 patients) presented as an expansive and osteolytic bone destruction with a soft tissue mass. Type II (2 patients) presented as a scalp mass with mild erosion of the outer skull plate. Type III (3 patients) presented as a scalp mass without skull destruction. All patients underwent surgical resection. For type I patients, craniectomy and cranioplasty were performed. For type II patients, complete excision of the scalp mass with local skull curettage was performed. For type III patients, complete excision of the scalp mass was performed. There were no cases of recurrence after follow-up.

**Conclusions:**

CF usually presents as a painless, firm, nonmobile and single mass with a clear boundary. There are generally three types of MSCT findings: bone destruction with a soft tissue mass, a scalp mass with erosion of the skull and a scalp mass. Different management strategies should be utilized for the various types of CF.

## Background

CF is a rare benign fibroproliferative lesion of the skull that almost exclusively affects children [1, 2]. The earliest description of CF dates to 1980 in a report by Lauer and Enzinger [3]. Commonly, CF is caused by post-traumatic, radiation therapy, hereditary and idiopathic conditions [4], and is presented histologically by fibroblasts and myofibroblasts with a varying collagenous and myxoid stromal background [5]. However, CF is difficult to diagnose by X-ray and multi-slice computed tomography (MSCT) before surgery due to the lack of knowledge of the imaging features. To date, approximately 80 CF cases have been reported [4]. Some of these patients have presented with imaging features in some case reports [4, 6, 7]. To the best of our knowledge, the summarized radiologic characteristics and the subtype of the disease have not been shown. Therefore, our purpose was to analyse the CF subtype and imaging presentation characteristics in 14 confirmed CF patients. In addition, all the patients underwent surgical resection of the lesion at our institution. Thus, the management and outcome of the disease were also evaluated in our study.

## Methods

### Patients

This retrospective study was approved by the institutional review board of Hunan Children’s Hospital, and the requirement for informed consent was waived. We searched our institution’s database to retrieve the pathologic, radiologic, and clinical data of patients with histologically confirmed CF who underwent an operation between January 2016 and October 2021. Consecutive patients with pathologically confirmed CF were identified and enrolled based on the following inclusion criteria: (1) CF confirmed by surgical pathology with a detailed pathological report; (2) MSCT imaging performed within two weeks before surgery; and (3) sufficient image quality to allow accurate interpretation of radiologic features. Patients were excluded if they had one or more of the following issues: (1) unavailability of MSCT imaging or imaging was performed at another institution; (2) suboptimal image quality making the evaluation of imaging characteristics difficult; and (3) previous surgical therapy. A total of 14 out of the 16 patients with CF were enrolled.

### MSCT examinations and image analysis

Fourteen patients underwent a plain MSCT scan of the head, and 4 patients underwent CT enhancement examination. MSCT examinations were performed with a Philips Brilliance 64 scanner. The imaging parameters were as follows: tube voltage 80–100 kV, tube current 80–120 mAs, scan thickness 0.625 mm, and axial scanning. Iodixanol (320 mg I/ml) was used for enhanced examination, and the dose was 2 ml /kg body weight. All images of patients were transferred to an imaging workstation (Philips Brilliance V4.5.4.50030) for postprocessing. The main methods of image processing were sagittal, coronal position and volume rendering (VR) to distinctly present the lesions. Two paediatric radiologists (YH.X., with 20 years of experience, and K.J., with 25 years of experience) reviewed all the images (including postprocessing images) with a consensus for each subject retrospectively. The image assessment included the position of the lesion (site of origin), size, radiologic characteristic and classification. For the soft tissue mass in every patient, the CT value was measured on plain and enhanced MSCT scans. The CT value was measured three times and averaged. The enhancement degree of the mass was evaluated with the following criteria: (1) marked, higher than 50 Hu; (2) moderate, higher between 20–50 Hu; and (3) weak enhancement, less than 20 Hu with comparison of the enhancement and plain phases. Microscope images equipment: LEICA DM 2000. Acquisition software: LOGENE Digital Imaging System (China). The image resolution:2048*1536.

### Treatment and follow-up

All patients underwent surgical resection of the lesion in our institution. All patients were followed up by telephone. The beginning time of the follow-up period was the time of surgery, and the deadline for inclusion was December 2021. The follow-up time ranged from 3 to 68 months, with an average time of 38 months.

## Results

### Clinical findings

A total of 14 CF patients (aged 5–107 months; average age 41.7 months; 6 males and 8 females) were included, and the clinical characteristics are summarized in Table 1. Of the 14 patients, only 2 patients (14.3%) presented tenderness at the lesion position, and 12 patients (85.7%) had a painless mass. Twelve patients (85.7%) presented firm and nonmobile masses, and 2 (14.3%) patients presented moderate hard and mobile masses. In the short term (1 to 2 months), the mass was clearly increased in 2 patients (14.3%), gradually increased in 5 patients (35.7%), and did not distinctly change in 7 patients (50.0%). In 12 patients (85.7%), only a single lesion appeared, and 2 patients (14.3%) displayed multiple lesions. A total of 18 lesions were detected in 14 patients, including 7 lesions in the parietal region, 5 lesions in the frontal and occipital regions, and only 1 lesion in the temporal region. The maximum diameter of the lesions ranged from 12 to 43 mm, and 13 lesions (13/18, 72.2%) were less than 20 mm. The white blood cell count, platelet count, coagulation and biochemical parameters of liver and kidney function were normal for all the patients. All the patients did not exhibit any neurologic impairment.

**Table 1:** Clinical features and treatment of 14 patients with CF

Case	Site of lesion	MD (cm)	Num	Presentation	Treatment	Follow up (months)
1	Right parietal	3.0	1	No growing, nontender, firm, and nonmobile mass	Craniectomy and cranioplasty	68
2	Occipital	2.0	1	Gradually growing, nontender, moderate hard, and mobile mass	Excision of scalp mass	65
3	Right temporal	2.7	1	Rapidly growing, nontender, firm, and nonmobile mass	Craniectomy and cranioplasty	63
4	Frontal	1.2	1	No growing, nontender, firm, and nonmobile mass	Craniectomy	51
5	Left parietal	4.3	1	No growing, nontender, firm, and nonmobile mass	Craniectomy and cranioplasty	43
6	Occipital	4.2	1	Gradually growing, nontender, moderate hard, and mobile mass	Excision of scalp mass	42
7	Right parietal	2.0	1	No growing, tenderness, firm, and nonmobile mass	Excision of scalp mass and skull curettage	41
8	Left parietal	1.4	1	Gradually growing, nontender, firm, and nonmobile mass	Craniectomy	40
9	Frontal	1.4	1	No growing, nontender, firm, and nonmobile mass	Excision of scalp mass and skull curettage	30
10	Frontal	3.1	1	Rapidly growing, tenderness, firm, and nonmobile mass	Craniectomy and cranioplasty	28
11	Occipital	1.2	3	Gradually growing, nontender, firm, and nonmobile mass	Excision of scalp mass	27
12	Right parietal	1.5	1	No growing, nontender, firm, and nonmobile mass	Craniectomy	22
13	Right parietal	1.2	1	No growing, nontender, firm, and nonmobile mass	Craniectomy	8
14	Frontal and right parietal	1.8	3	Gradually growing, nontender, firm, and nonmobile mass	craniectomy and cranioplasty	3

*MD* maximum diameter of the lesion, *Num* the number of lesions in one patient

### MSCT findings and subtype

**Fig. 1:**
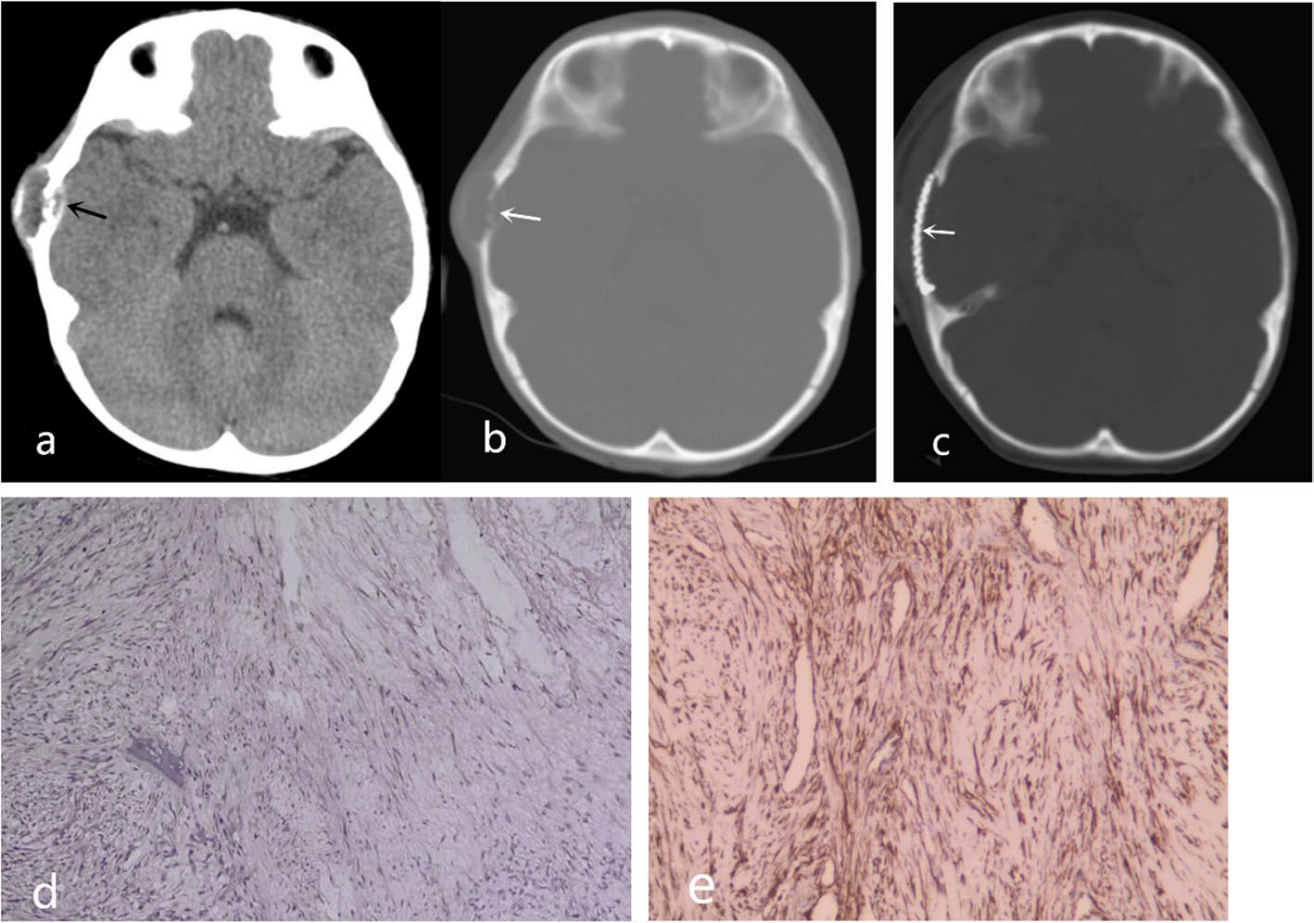
Type I. Expansive and osteolytic bone destruction of the right temporal with iso-density soft tissue mass was shown, and the lesion penetrated through the cranial plate involving the dura (**a**, arrow). Residual bone fragments in the lesion were observed (**b**, arrow). Craniectomy and cranioplasty were performed (**c**, arrow). Histopathologic examination (H&E stain, × 100) of the specimen showed proliferation of spindled fibroblasts/myofibroblasts are arranged in bundles, and the myxoid stroma was loose and edema (**d**). Immunohistochemistry displays positive SMA staining (e, smooth muscle actin, × 200)

**Fig. 2:**
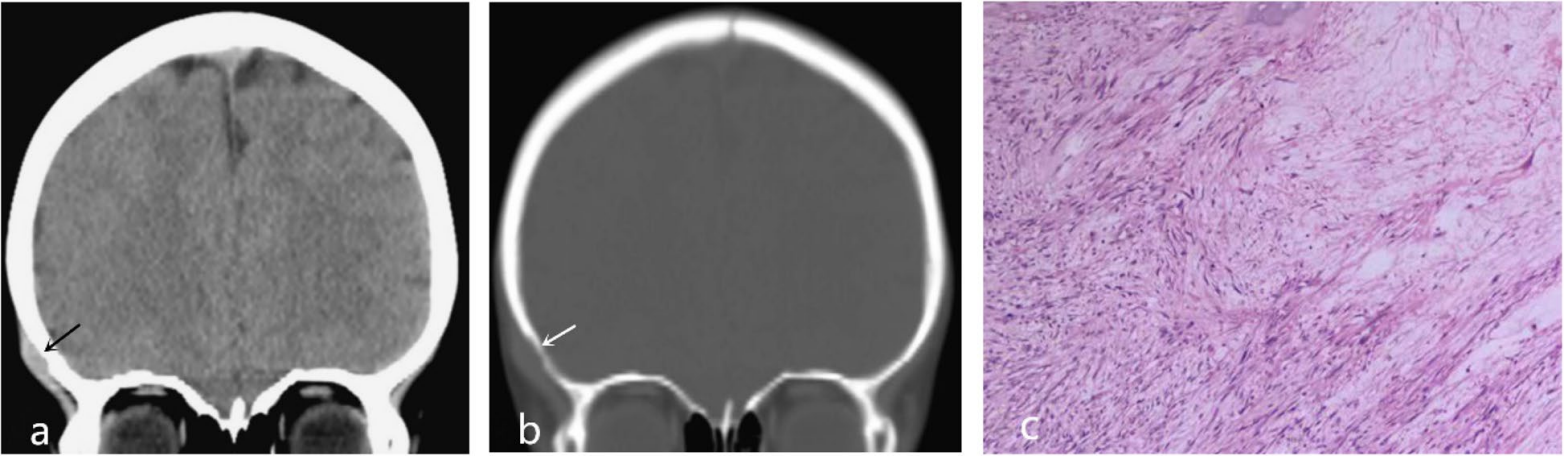
TypeII. A scalp mass was observed in the right frontotemporal region (**a**, arrow), with mild erosion of the outer skull plate (**b**, arrow). Histopathologic examination (H&E stain, × 100) of the specimen showed proliferation of spindled fibroblasts/myofibroblasts are arranged in bundles (**c**)

**Fig. 3:**
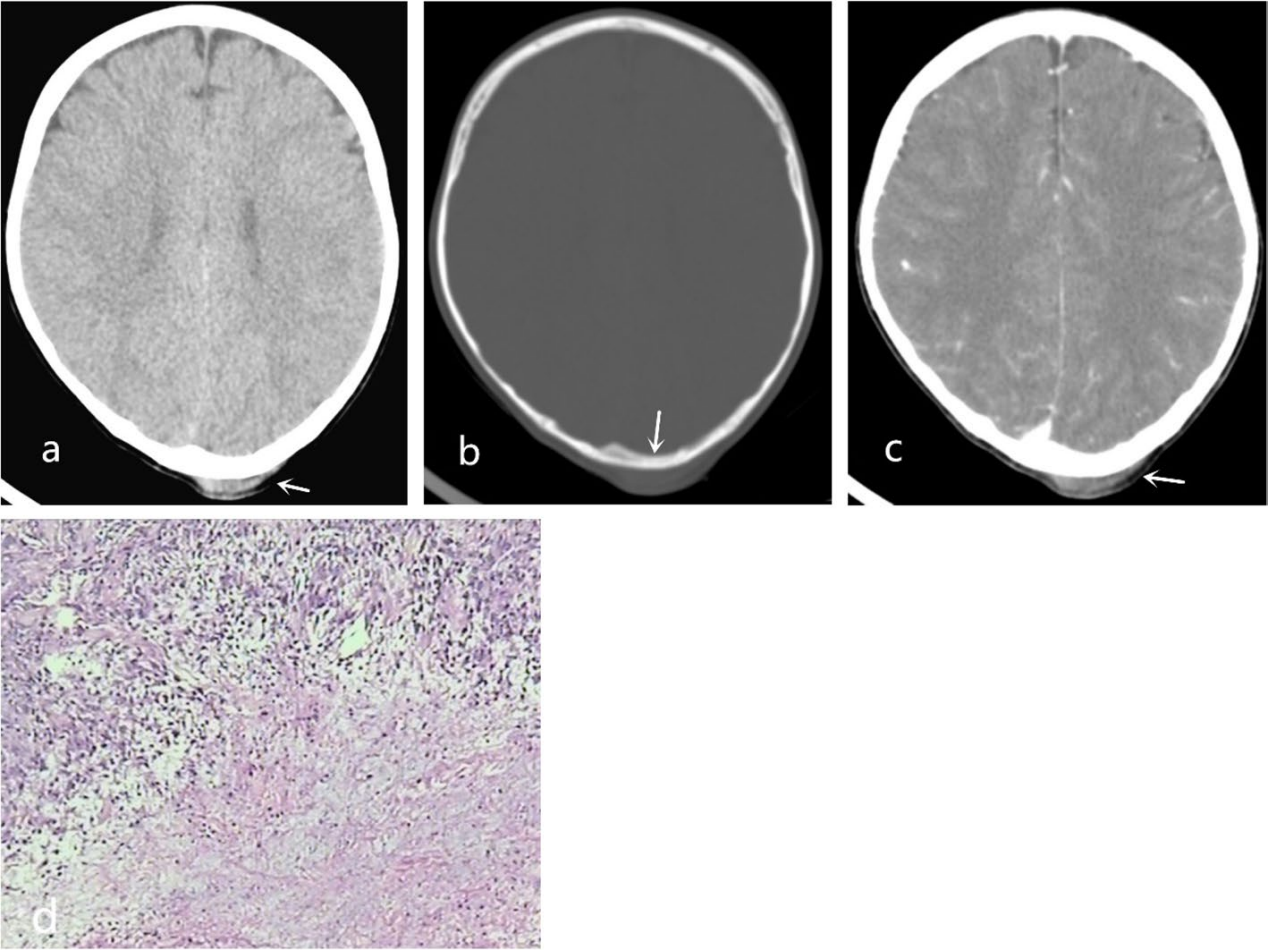
Type III. A scalp mass was observed in the occipital region (**a**, arrow), with normal skull (**b**, arrow). Enhanced CT showed mild enhancement of the scalp mass (**c**, arrow). Histopathologic examination (H&E stain, × 100) showed proliferation of spindled fibroblasts/myofibroblasts (**d**)

The imaging findings of 14 CF patients are summarized in Table 2. We categorized these patients into three types (type I, II and III) according to whether the soft mass involved the skull and combined with bone destruction in MSCT characteristic appearance. The characteristic features of type I (9 patients, 64.3%) presented as an expansive and osteolytic bone destruction with soft tissue mass (Fig. 1). For 8 of the patients, the lesion penetrated through the cranial plate involving the dura. Type II (2 patients, 14.3%) presented as a scalp mass with mild erosion of the outer skull plate (Fig. 2). Type III (3 patients, 21.4%) presented as a scalp mass without skull destruction (Fig. 3). A soft tissue mass was found in all 14 patients, which was usually shown on plain CT as an isodensity or slight high density with a clear boundary. Mild or no sclerosis was shown at the edge of bone destruction. Four patients underwent contrast-enhanced CT, of which 2 patients showed obvious enhancement, 1 showed mild enhancement, and 1 showed moderate enhancement. The cranial suture was affected in 4 patients. All patients were not diagnosed with CF by a radiologist before surgery.

**Table 2:** CT findings and classification of 14 patients with CF

Case	CT features	Invasion	ICS	RB	Plain CT	CE-CT	Type
1	Lytic destruction with soft tissue mass	Penetration through the cranial plate and involving the dura	No	Yes	44 Hu, uniform	NP	I
2	Scalp mass with normal skull	No penetration through the cranial plate	No	NA	45 Hu, uniform	NP	III
3	Lytic destruction with soft tissue mass	Penetration through the cranial plate and involving the dura	No	Yes	35 Hu, uneven	NP	I
4	Expansive, lytic destruction with soft tissue mass	Penetration through the cranial plate and involving the dura	No	Yes	46 Hu, uniform	97Hu, uniform	I
5	Expansive, lytic destruction with soft tissue mass	Destroyed diploe and erosion of the inner and outer skull plates	No	Yes	40 Hu, uneven	67Hu, uneven	I
6	Scalp mass with normal skull	No penetration through the cranial plate	No	NA	37 Hu, uniform	49Hu, uniform	III
7	Scalp mass with eroded outer plate of skull	No penetration through the cranial plate	No	NA	50 Hu, uniform	NP	II
8	Expansive, lytic destruction with soft tissue mass	Penetration through the cranial plate and involving the dura	Yes	Yes	42 Hu, uneven	NP	I
9	Scalp mass with eroded outer plate of skull	No penetration through the cranial plate	Yes	NA	40 Hu, uneven	NP	II
10	Expansive, lytic destruction with soft tissue mass	Penetration through the cranial plate and involving the dura	Yes	Yes	35 Hu, uneven	92Hu, uneven	I
11	Scalp mass with skull compression	No penetration through the cranial plate	No	NA	43 Hu, uniform	NP	III
12	Expansive, lytic destruction with soft tissue mass	Penetration through the inner skull plate and involving the dura	No	Yes	36 Hu, uniform	NP	I
13	Expansive, lytic destruction with soft tissue mass	Penetration through the cranial plate and involving the dura	Yes	Yes	37 Hu, uneven	NP	I
14	Expansive, lytic destruction with soft tissue mass	Penetration through the cranial plate and involving the dura	No	Yes	40 Hu, uniform	NP	I

*ICS* invade cranial suture, *RB* residual bone in the lesion, *NP* not performed, *NA* not applicable, *CE* contrast enhancement

### Management

All patients underwent surgical resection. For type I patients, craniectomy was performed in 9 patients, and additional cranioplasty was performed in 5 patients due to the larger lesion. For type II patients, complete excision of the scalp mass with local skull curettage was performed in 2 patients. For type III patients, complete excision of the scalp mass was performed in 3 patients. There were no cases of recurrence during the follow-up period.

## Discussion

### Clinical features

In our CF patients, the male to female ratio was 6:8 which is different from the previously reported male predominance among patients with CF [4]. However, this may be related to the small number of our patients. The temporal and parietal regions are the most commonly affected areas of CF [8], but they can occur in any location of the skull, even presenting as an intracranial mass [9, 10]. In this study, a total of 18 lesions were detected in 14 patients, including 7 lesions at the parietal, 5 lesions at the frontal and occipital regions, and only 1 lesion at the temporal region. Most CF lesions are single, and multiple lesions are rarely reported [11, 12]. In this group, 2 patients had multiple lesions, one of which had three scalp masses at the occipital without destruction of the skull, and the other patient had two lesions in the frontal bone and one lesion in the right parietal bone. The typical clinical manifestations of CF are usually a painless, palpable scalp mass without obvious discomfort and few neurological symptoms or signs. The clinical symptoms of CF have a certain relationship with the location and depth of invasion of the lesions, which can appear as eyeball protrusion, diplopia, facial paralysis, deafness and hemiplegia [9, 13]. All of the patients in this study were brought to our hospital due to an accidental discovery of a head mass. Two patients had tenderness at the site of the mass, but all patients had no other complaints. In the short term, the masses of 2 patients grew rapidly, and those of 5 patients grew gradually. Although some investigators evaluated systemic serum inflammatory markers, such as the white blood cell count, C-reactive protein, platelet count and erythrocyte sedimentation rate, the values were within the normal range, indicating that CF is likely a localized rather than a systemic inflammatory or reactive process [12]. In this study, the white blood cell count, platelet count, coagulation and biochemical parameters of liver and kidney function were normal for all the patients.

### CT findings

Imaging is usually employed to further characterize lesions after the initial examination. Our study showed bone involvement in 78.6% of CF patients. A CT scan can be used to evaluate the extent of bone involvement, and the findings can be helpful in surgical planning. In this study, we divided the imaging manifestations of CF into three types according to whether the soft mass involved the skull and combined with bone destruction in the characteristic MSCT appearance. Type I manifestations had severe bone destruction and were characterized by an expansive and osteolytic bone destruction with a soft tissue mass. There was mild or no sclerosis at the edge of bone destruction, and the lesion usually penetrated through the cranial plate involving the dura. This type was the most common accounting for 64.3% in our study. The same CT features of patients with CF have been described in many previous case reports [1, 4, 14–17]. In addition, we noted that residual bone in soft tissue masses was usually observed. We believe that these imaging characteristics are important in diagnosing and distinguishing CF from other skull lesions. Type II manifestations (14.3%) are characterized by a scalp mass with mild erosion of the outer skull plate, and the inner plate of the skull remained intact. Type III manifestations (21.4%) presented as a scalp mass without skull destruction, although the skull can be compressed. Type III manifestations have been described in a few previous reports, but type II manifestations have rarely been reported [4, 11]. We speculate that type II manifestations may be the early stage of type I or the late stage of type III, or that the lesion origin of type II manifestations may be different from the other two types. Previous reports have shown that the imaging findings of soft tissue masses are varied, and their density or signal can be homogeneous or heterogeneous, which can range from no enhancement to a significant enhancement [4, 12, 16]. In our patients, soft tissue masses usually showed an iso-density or slight high density with a clear boundary on the plain CT scan, and the density was uniform in 11 patients. Among the 4 patients who underwent enhancement examination, 2 patients presented obvious enhancement, 1 patient presented mild enhancement, and 1 patient presented moderate enhancement. The lesions were usually small, and the maximum diameter of 72% of the lesions was less than 2 cm in this study. The cranial suture could be invaded by lesions, and there were 4 patients in this group in which the cranial suture was involved.

### Management

Surgical resection of the lesion is recommended for the treatment of CF [4, 11, 18]. In our study, all the patients underwent surgical resection of the lesion. For 9 patients with type I lesions, the soft tissue mass and destroyed skull were completely excised. Due to the large skull defect after excision, additional cranioplasty with titanium mesh was performed in 5 of the patients with large lesions. A report suggests that large skull defects after excision in infants can be reconstructed with autograft-harvested bone from the rib or split calvarial bone [1], but more studies of this surgery are necessary. Dura adhesion was found in 8 patients during the operation, which was consistent with the CT findings. For 2 patients with type II lesions, complete excision of the scalp mass with local eroded skull curettage was performed. For patients with type I and type II lesions, we suggest that an incisional biopsy is necessary to rule out a malignant process. For 3 patients with type III lesions, complete excision of the scalp mass was performed. The skulls were found to be intact during surgery, although in one patient, CT showed skull compression. Except for surgical resection, a case report described an alternative treatment using intralesional triamcinolone acetonide injection with complete resolution of a lesion in the occipital bone [19]. Radiotherapy and chemotherapy are not recommended. Recurrence following excision is uncommon. The total recurrence rate for CF after resection was 7.5%. The recurrence rate was 15.7% for those treated with curettage or no bone resection and was 6% in those who had undergone a full craniectomy [4]. No recurrence was found in any of our patients during follow-up, which might indicate that aggressive resection could reduce the recurrence rates, although the patient numbers were too low to determine statistical significance.

### Differential diagnosis

CF represents a rare type of skull lesion, which is usually not considered in differential diagnosis by radiologists due to the deficient knowledge of the clinical presentation and imaging features. None of the patients in our study were diagnosed with CF before surgery. CF with a type I lesion is usually misdiagnosed as Langerhans cell histiocytosis (LCH), because the CT findings of cranial LCH mainly also show osteolytic bone destruction with a soft tissue mass [20]. In addition, the clinical course characterized by a rapidly growing lesion and the invasive nature of CF with type I and type II lesions can make it difficult to distinguish from malignant conditions, such as sarcoma. For type III lesions, a differential diagnosis should include other conditions, such as fibroma, haemangioma, epidermoid cyst and haematoma, that produce an enlarging scalp mass in a paediatric patient.

### Limitations

The limitations of this study are the small number of CF patients, retrospective design, and single-centre design. No MRI images were available for feature analysis. With advances in imaging modalities and the rising popularity of health examinations, diagnosis of CF will increase, thus promoting better treatment plans for patient management.

## Conclusions

CF is a rare benign hyperplastic lesion involving the scalp and skull that usually presents as a painless, firm, nonmobile, growing and single mass with a clear boundary. There are generally three types of MSCT findings: expansive and osteolytic bone destruction with soft tissue mass, scalp mass with skull erosion and scalp mass without skull destruction. Different management strategies should be performed for patients with the various types of CF on MSCT, which is helpful to the benefit children with CF.

## Data Availability

The datasets used and/or analysed during the current study are available from the corresponding author on reasonable request.
